# Application of Box-Behnken experimental design and response surface methodology for selecting the optimum RP-HPLC conditions for the simultaneous determination of methocarbamol, indomethacin and betamethasone in their pharmaceutical dosage form

**DOI:** 10.1186/s13065-022-00908-9

**Published:** 2022-12-12

**Authors:** Ehab F. Elkady, Marwa A. Fouad, Ayoub N. Mozayad

**Affiliations:** 1 Pharmaceutical Chemistry Department, Faculty of Pharmacy, Cairo University, Kasr El-Aini Street, Cairo, 11562 Egypt; 2 Pharmaceutical Chemistry Department, School of Pharmacy, NewGiza University, Newgiza, km 22 Cairo– Alexandria Desert Road, Cairo, Egypt; 3 Pharmaceutical Chemistry Department, Faculty of Pharmacy, Sana’a University, Sana’a, Yemen

**Keywords:** Methocarbamol, Indomethacin, Betamethasone, Box-Behnken design, RP-HPLC, Response surface methodology

## Abstract

An isocratic RP-HPLC method has been developed for the separation and determination of methocarbamol (MTL), indomethacin (IND), and betamethasone (BET) in combined dosage form using an Inertsil ODS-3v C18 (250 × 4.6 mm, 5 μm) column with UV- detection at 235 nm. Experimental design using Box-Behnken design (BBD) was applied to study the response surface during method optimization and to achieve a good separation with a minimum number of experimental runs. The three independent parameters were pH of buffer, % of acetonitrile and flow rate of the mobile phase while the peak resolution of IND from MTL and the peak resolution of BET from IND (R2) were taken as responses to obtain mathematical models. The composite desirability was employed to optimize a set of responses overall (peak resolutions). The predicted optimum assay conditions include a mobile phase composition of acetonitrile and phosphate buffer (pH 5.95) in a ratio of 79:21, *v/v*, pumped at a flow rate of 1.4 mL min^−1^. With this ideal condition, the optimized method was able to achieve baseline separation of the three drugs with good resolution and a total run time of less than 7 min. The linearity of MTL, IND, and BET was determined in the concentration ranges of 5–600 µg mL^− 1^, 5–300 µg mL^− 1^, and 5–300 µg mL^− 1^ and the regression coefficients were 0.9994, 0.9998, and 0.9998, respectively. The average percent recoveries for the accuracy were determined to be 100.41 ± 0.60%, 100.86 ± 0.86%, and 100.99 ± 0.65% for MTL, IND, and BET, respectively. The R.S.D.% of the intra-day precision was found to be less than 1%, while the R.S.D.% of the inter-day precision was found to be less than 2%. The RP-HPLC method was fully validated with regard to linearity, accuracy, precision, specificity, and robustness as per ICH recommendations. The proposed method has various applications in quality control and routine analysis of the investigated drugs in their pharmaceutical dosage forms and laboratory-prepared mixtures with the goal of reducing laboratory waste, analysis time, and effort.

## Introduction

**Fig. 1:**
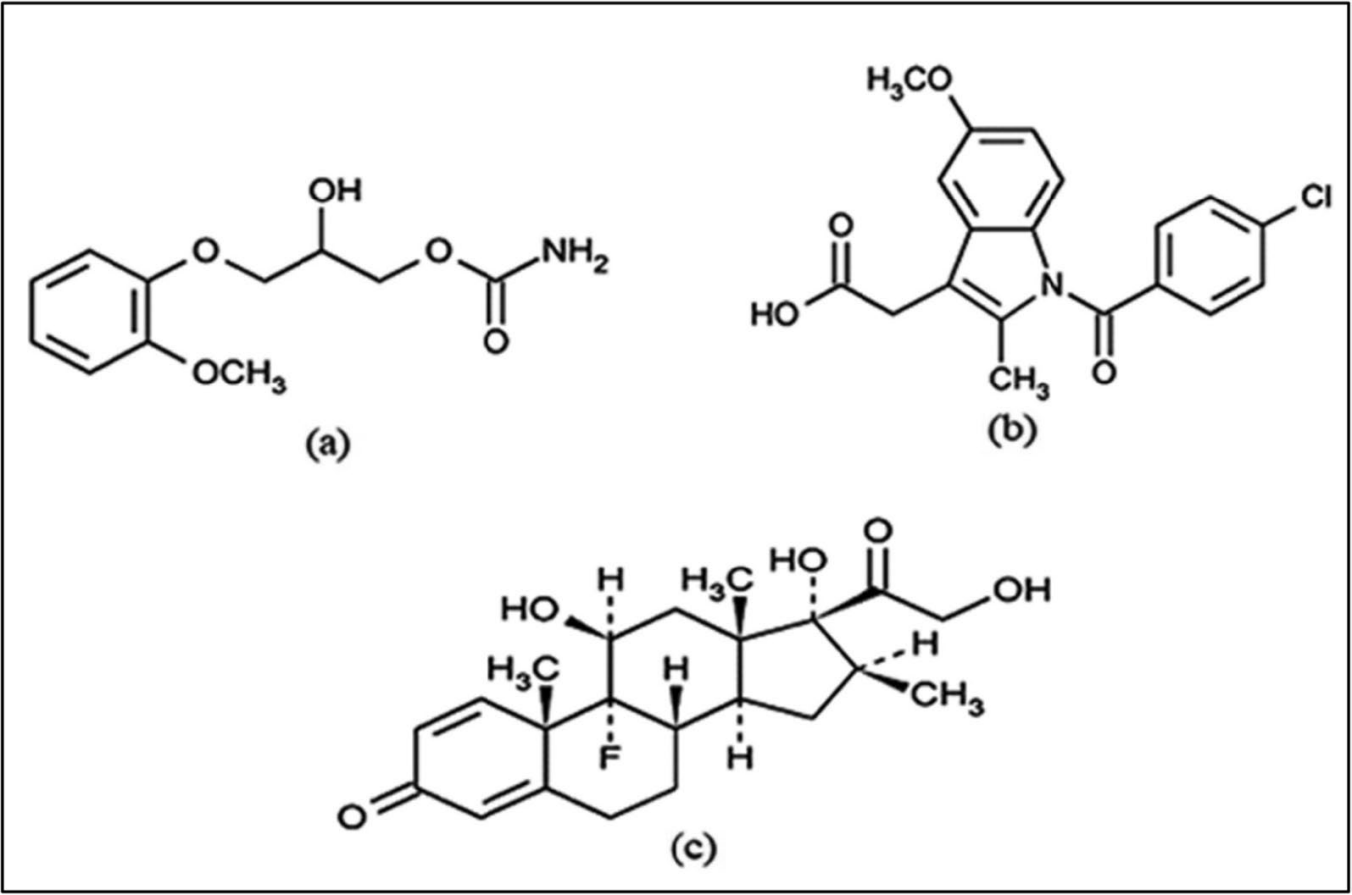
Chemical structures of **a** methocarbamol, **b** indomethacin and **c** betamethasone

Methocarbamol (MTL) is a muscle relaxant that acts centrally, chemically designated as (±)-3-(o-methoxyphenoxy)-1,2-propanediol-1-carbamate, Fig. 1a [1], which is used for the treatment of skeletal muscle spasms [2]. Indomethacin (IND) is an indole acetic acid derivative of nonsteroidal anti-inflammatory drugs, chemically designated as 1-(p-chlorobenzoyl)-5-methoxy-2-methylindole-3-acetic acid, Fig. 1b [1]. IND has anti-inflammatory, analgesic, and antipyretic activity. It has a potent inhibitory action on the cyclooxygenase enzymes, reduces the synthetic process of prostaglandins, and is used for the symptomatic treatment of musculoskeletal disorders such as rheumatoid arthritis, osteoarthritis, gouty arthritis, and disorders of the collagen alone or in combination with other drugs [3–5]. Betamethasone (BET), chemically designated as 9-fluoro-11β,17,21-trihydroxy-16β-methylpregna-1,4-diene-3,20-dione, Fig. 1c [1], is a long-acting synthetic fluorinated glucocorticosteroid that can be administered orally, parenterally, or topically in the management of various disorders such as acute gouty arthritis due to its anti-inflammatory and analgesic effects.

The first commercially available HPLC system was developed in 1969 as a result of the development of columns and online detectors. Since that time, HPLC has become a good instrument for chromatographic analyses. Today, HPLC has achieved a great reputation in separation science because of its high speed, selectivity, efficiency, low detection and quantification limits, and reproducibility [6, 7]. Because of its ability to separate quite complex mixtures of low and high molecular weight compounds, as well as different polarities and acid-base properties in various matrices, it is also a well-known and well-established separation technique that is frequently used to resolve a variety of analytical problems [8]. Despite the wide variety of drug analysis techniques that are available, including titrimetry, spectroscopy, and electroanalysis, HPLC is still the technique that is most frequently employed in research centers, academic labs, or industrial companies for pharmaceutical analysis [9].

Various HPLC methods were published for determining MTL either alone or in combination with other drugs in pharmaceutical preparations in the last 10 years [2, 10–19]. IND was determined by HPLC individually or simultaneously with other drugs in pharmaceutical dosage forms [3–5, 20]. BET was determined by using HPLC either alone or with other drugs [21–25]. Although some HPLC methods were described for determining BET esters individually or in combination with other substances in the last ten years [26–34], no methods were reported for the simultaneous determination of MTL, IND, and BET in capsules dosage forms. In addition, some of the reported methods required particular columns [26, 35], more expensive detectors that might not be available in all laboratories [10, 36], and some of them had narrower linearity ranges [5, 10, 11, 16, 17, 23, 25, 27–29, 31, 33, 36–39] and longer analysis times than the proposed method [3, 4, 17, 21, 28, 32, 36–38, 40–42]. Additionally, the proposed method was found to have larger linearity ranges, higher precision, and greater sensitivity than some other reported analytical methods and was much simpler in its mobile phase composition compared to the reported gradient elution methods [2, 12, 17, 21, 28, 29, 31].

This study describes the optimization and validation of the HPLC method for the analysis of MTL, IND, and BET in capsules dosage forms using response surface methodology (RSM) to facilitate the optimization step of the experimental conditions which is a very complex step because of the large number of parameters that must be simultaneously tested [43]. RSM is a group of mathematical and statistical techniques that are used for improving and optimizing analytical methods and are commonly used to get the best chromatographic parameters for separation and determination of chemical components. RSM provides many benefits over the traditional “one-variable-at-a-time” optimization method, which is time-consuming and depends on a huge number of experimental trials to get the best ideal chromatographic conditions. Firstly, RSM provides an enormous amount of information from a small number of experimental trials. Secondly, RSM easily distinguishes the interaction effect among the independent and dependent predictors. The regression model equation easily describes the effects of independent predictors. Furthermore, the empirical model that describes the relationship among the independent and dependent predictors is used to get some data about the process and is also used in the prediction process. According to the aforementioned aspects, RSM is an effective technique for the optimization and prediction processes. Finally, RSM is a cost-effective technique, as the number of experimental runs can be greatly decreased [44–46].

Thus, the goal of this work was to apply the response surface methodology involving Box–Behnken design to develop and optimize an accurate, precise, specific, and robust RP-HPLC method for the separation and simultaneous determination of MTL, IND, and BET, which has a variety of uses and can be applied in routine and quality control analysis of the cited drugs in both pure or combined dosage forms to save time and reduce effort, cost, and laboratory waste. Furthermore, the robustness of the reported method was investigated by utilizing the constructed models to save time and money. Additionally, method validation was carried out in accordance with ICH requirements Q2 [47]. The novelty of this study concerns the application of the RP-HPLC method for the simultaneous separation and determination of the three drugs. Despite the fact that many reported studies determined these drugs individually or in combination with other drugs, no methods were reported for simultaneous determination of the three drugs. The importance of this study lies in its ability to determine the quantity of these three drugs despite the presence of a large difference in the ratio between the quantity of these drugs as well as the estimation of the quantity of betamethasone, which is found in a very small amount compared to the rest of the drugs.

## Experimental

### Instrument

An Agilent HPLC system 1100 series (Japan) equipped with a G1314A variable wavelength detector and G1310A Iso pump was used for the chromatographic separation. Agilent Chemstation PC software was employed for the instrumental control, data analysis, and acquisition. Separation was done using a Inertsil ODS-3*v* C18 column (250 mm, 4.6 mm, 5 μm). A pH meter (Jenway, 3505, UK) was employed for adjustment of pH. A sonicator (Soniclean-120T, The barton-SA, Australia) was employed. 0.45 μm membrane filters from Sartorius Stedim-Biotech GmbH (Goettingen, Germany) were employed for mobile phase filtration. Minitab-17 (Minitab, Inc., State College, PA, USA) was employed for analysis of data and experimental design [48].

### Materials and substances

MTL, IND, and BET standards were generously provided from October Pharma (6 October City, Egypt), Misr Pharma (Cairo, Egypt), and Amoun Pharmaceutical Company (Cairo, Egypt), respectively. Purities were 99.50%, 100.50%, and 99.60%, respectively. From the Mexican market, Ardosons^®^ capsules containing 215 mg MTL, 25 mg IND, and 0.75 mg BET were acquired. Aquatron Water Still was used to obtain bidistilled water (A4000D, Staffordshire, UK). *o*-Phosphoric acid and HPLC-grade acetonitrile were purchased from Sigma-Aldrich (Darmstadt, Germany). Monobasic potassium phosphate purchased from Loba Chemie Ltd. (Mumbai, India) was employed for preparing aqueous buffer solutions.

### Chromatographic procedure

The HPLC separation and determination were performed on an Inertsil-ODS-3*v* C18 column (250 mm x 4.6 i.d., 5 μm). The mobile phase consisted of acetonitrile and monobasic potassium phosphate (50 mM, adjusted to pH 5.95 with sodium hydroxide) (79:21, *v/v*). After passing through a 0.45 μm membrane filter, the mobile phase was degassed using an ultrasonic bath and pumped at a 1.4 mL min^− 1^ flow rate. The peak area was used for quantification, with UV-estimation at 235 nm.

### Preparation of standard stock solutions

An accurate weight of 215 mg of MTL, 250 mg of IND, and 75 mg of BET were separately weighed and transferred into a series of 100 mL volumetric flasks. The contents of the flasks were dissolved in the mobile phase and completed to volume to obtain 2.15 mg mL^− 1^ of MTL, 2.5 mg mL^− 1^ of IND, and 0.75 mg mL^− 1^ of BET stock solutions. During the analysis, freshly prepared working solutions were created by diluting stock solutions.

### Sample preparation

The contents of 10 Ardosons^®^ capsules were weighed and mixed in a mortar, and an amount of well-mixed powder equivalent to the contents of four Ardosons^®^ capsules was accurately put into a 100 mL volumetric flask. Sufficient volume of mobile phase (50 mL) was added, and the flask was well shaken and sonicated for at least 15 min. Flask volume was completed with the mobile phase to the mark, and the sample stock solution (8600 µg mL^− 1^ MTL, 1000 µg mL^− 1^ IND and 30 µg mL^− 1^ BET) was obtained then it was filtered through Whatman filter paper and discarding a few mL of the filtrate. After filtration, different aliquots from sample stock solution were transferred to 2 series of 10 mL volumetric flasks and then completed with the mobile phase to the volume for the determination of MTL, IND, and BET.

### Experimental design for optimizing chromatographic parameters

The critical parameters and their levels (low and high) for the experimental design were determined through preliminary experiments. The following parameters were examined in this step: chromatographic column type; buffer type and pH; mobile phase organic modifier (acetonitrile or methanol); percentage of organic modifier; and flow rate. Based on the preliminary results, a three-factors, three-levels BBD with three repeated runs at the center point, was chosen to optimize the separation of the three drugs with good resolution. By mapping the chromatographic response surface, BBD was used to optimize the primary parameters affecting HPLC resolution. Fifteen runs were designated and then experimentally carried out using the run order shown in Table 1. The chosen factors were pH of buffer, % of acetonitrile, and flow rate, and the peak resolutions of IND from MTL [49] and BET from IND (R2) were measured as responses. At three levels, each factor was tested. The experimental results were fitted with a second-order model. The coefficient of determination R, the probability values (P < 0.05), and the p-value for lack-of-fit were applied to assess the quality of fitted polynomial models. By applying Derringer’s desirability function (D), the true position of the optimal condition was determined, where responses were simultaneously optimized, and finally the design space of responses was predicted using the regression model equation.

**Table 1:** A Box-Behnken design data matrix and responses

Design points	Factor levels			Responses	
Sample runs	pH	F (mL min^− 1^)	Ratio	R1	R2
1	5.3	1.2	76	10.39	7.06
2	5.3	1.3	72	11.26	6.85
3	6.3	1.2	76	7.74	10.50
4	5.8	1.4	80	8.37	6.41
5	5.8	1.2	72	10.60	8.74
6	5.8	1.3	76	9.23	8.26
7	6.3	1.3	80	7.28	8.86
8	5.8	1.2	80	8.87	6.44
9	5.3	1.4	76	9.68	5.49
10	5.8	1.3	76	9.69	8.14
11	6.3	1.3	72	6.63	11.30
12	5.8	1.4	72	10.00	8.37
13	5.8	1.3	76	9.59	8.04
14	6.3	1.4	76	6.69	10.94
15	5.3	1.3	80	8.42	4.57

pH = pH of buffer

F = flow rate of the mobile phase in mL min^− 1^

Ratio = % of Acetonitrile

R1 = resolution response between methocarbamol and indomethacin

R2 = resolution response between indomethacin and betamethasone

### Procedure

#### Linearity

Different concentrations of standard stock solutions corresponding to 50–6000 µg of MTL, 50–3000 µg of IND, and 50–3000 µg of BET were accurately measured and transferred into a series of 10 mL volumetric flasks. With the mobile phase, the volume was adjusted to the mark. Twenty µL were drawn from each flask and injected three times onto the column. The previously indicated chromatographic conditions were achieved. By applying the relationship between peak area (A) and the corresponding drug concentration (C), three calibration curves were plotted [50].

### Analysis of pure mixtures prepared in laboratory

Different aliquots of standard stock solutions of MTL, IND, and BET were transferred to a series of 10-mL volumetric flasks, and the volume was completed to the mark with the mobile phase to get concentrations in the range of 344–580 µg mL^− 1^ MET, 40–200 µg mL^− 1^ IND, and 12–150 µg mL^− 1^ BET. The process was carried out as described under the “Linearity” section. The concentrations of MTL, IND, and BET were estimated by applying the appropriate regression equations.

### Analysis of MTL, IND, and BET in capsules dosage forms

For determining MTL, IND, and BET in capsules, the stock sample solution was diluted with the mobile phase and then injected into the column in triplicates. To calculate the amounts of MTL, IND, and BET, the regression equations for each drug were used.

## Results & discussion

The fundamental goal of developing an RP-HPLC method for the concurrent quantification of MTL, IND, and BET is to separate them with a good resolution factor (RS > 2.0), acceptable peak symmetry, and adequate retention time, which can be obtained by changing one of the significant chromatographic parameters, such as the pH of the buffer, the % of acetonitrile, or the flow rate of the mobile phase [51, 52].

### Design of experiments to optimize chromatographic parameters

The design of experiments is a consecutive and complicated procedure because many factors can affect HPLC separation. It is used to construct and analyze experimental runs to identify the relationship among the dependent predictors and the main independent variable effects, as well as their interaction effects that produce an optimum response. A DoE approach using BB design was employed to construct the RSM that helps in discovering chromatographic parameters that give appropriate separation by using minimum experimental trials with minimum consumption of time and effort, as well as identifying the importance of these parameters and constructing regression models that produce polynomial second-order equations for the prediction process of responses [53]. BBD was utilized to optimize and assess the main effects, quadratic effects, and interaction effects of independent parameters on the interested responses, i.e., BBD considers the linear and quadratic effects, as well as interaction effects among the variables under investigation. The selected variables for investigation were pH of buffer (pH), % of acetonitrile (Ratio), and flow rate of mobile phase (F), where the three-levels (−1, 0, + 1) for each variable were chosen after preliminary trials. The three-levels for the selected variables and the number of experimental runs are shown in Table 1. The response estimation at the zero-level for each variable (0, 0, 0) was replicated three times through the runs to determine the pure errors, while the remaining 12 runs were randomly performed according to run orders to reduce the effects of uncontrolled factors that may introduce biased responses [44, 54]. The regression model analysis was applied to analyze the collected data and produce the relationship between the responses and the independent variables, which are represented by the following second-order polynomial equations R1 (Eq. 1) and R2 (Eq. 2):

1 $$R1 = 105.7+ 8.94 pH - 3.575 F - 2.701 Ratio - 3.871 pH*pH + 0.4356 pH*Ratio$$

2 $$R2 = -92.8- 26.35 pH- 60.06 F+ 5.673 Ratio+ 1.528 pH*pH- 0.03917 Ratio*Ratio+10.02pH*F$$

where R1 and R2 are the resolution responses between (MTL and IND) and (IND and BET), respectively. pH = pH of buffer, Ratio = % of acetonitrile, and F = flow rate. pH*Ratio and pH*F represent the interaction between the factors, while pH*pH is the quadratic term of buffer pH and Ratio*Ratio is the quadratic term of % of acetonitrile [55].

### Model statistical analysis

The models were statistically analyzed using the analysis of variance [56] test, and the results are shown in Table 2. The model and terms are significant when the probability P-value is less than 0.05. The R-sq (R-squared) and R-sq (adj) (adjusted- R-squared) values for the regression models were both within the acceptable limits (R > 0.8) that help in the estimation of model predictive power and show that the model is a good fit with a polynomial equation. High R-square and adjusted R-square values show adequate data fitting, whereas high predicted R-squared values reflect the model’s high prediction ability for new estimations [53]. Furthermore, the p-values for lack-of-fit commonly used to confirm that the model apparently represents the experimental results at a confidence limit of 95% are shown in Table 3.

**Table 2:** Analysis of variance [56] results for the models of MTL, IND and BET

Source of variation	Full quadratic models	
	R1 response	R2 response
	p-Value	p-Value
Constant	0.000	0.000
pH	0.000	0.000
F	0.002	0.005
Ratio	0.000	0.000
pH*pH	0.000	0.001
Ratio*Ratio		0.000
pH*Ratio	0.000	
pH*F		0.000
Residual error		
Lack of fit	0.621	0.380

pH = pH of buffer

F = flow rate of the mobile phase in mL min^− 1^

Ratio = % of Acetonitrile

R1 = resolution response between methocarbamol and indomethacin

R2 = resolution response between indomethacin and betamethasone

**Table 3:** Models fitting results

Model term	Full quadratic models	
	R1 response	R2 response
R-squared	98.29	99.70
Adjusted R-squared	97.34	99.47
Predicted R-squared	93.83	98.59
P-value of lack of fit	0.621	0.380

R1 = resolution response between methocarbamol and indomethacin

R2 = resolution response between indomethacin and betamethasone

### Effects of the factors

From the regression analysis of the models, the polynomial (second-order) equations determine the curvature in the relationship among the response variables (R) and the predictor variables. The coefficient value for each term estimates the change in the response variable per unit change in the predictor variable while keeping the other predictors in the model constant.

**Fig. 2:**
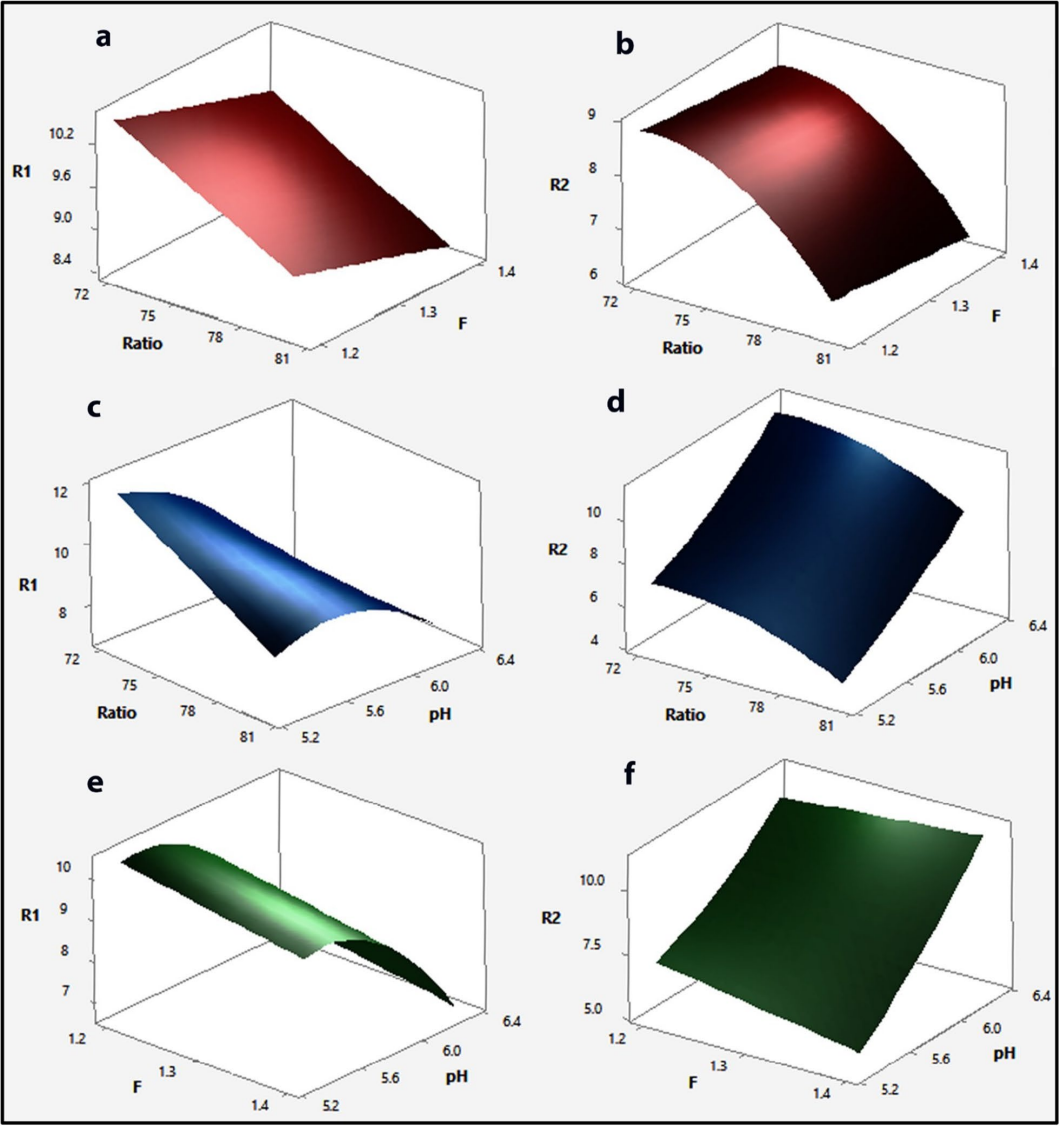
Three-dimensional 3D-response surface plots showing **a**, **b** the effects of Ratio (% of acetonitrile) and F (flow rate) on R1 and R2, respectively, **c**, **d** the effects of Ratio (% of acetonitrile) and pH of buffer on R1and R2, respectively, **e**, **f** the effects of F (flow rate) and pH of buffer on R1and R2, respectively. R1: resolution of indomethacin peak from methocarbamol peak. R2: resolution of betamethasone peak from indomethacin peak

**Fig. 3:**
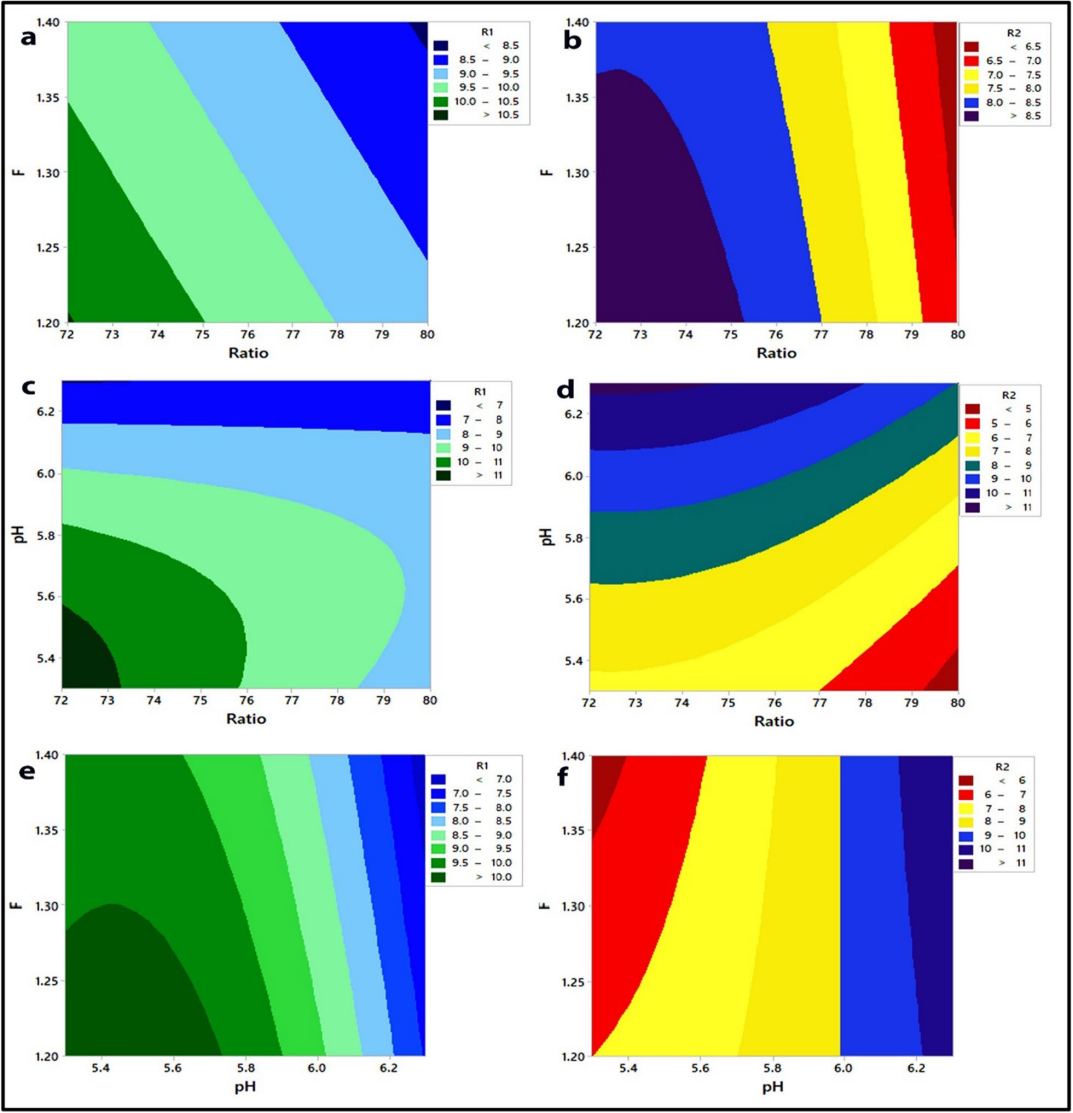
Contour plots showing **a**, **b** the effects of Ratio (% of acetonitrile) and F (flow rate) on R1 and R2, respectively, **c**, **d** the effects of Ratio (% of acetonitrile) and pH of buffer on R1and R2, respectively, **e**, **f** the effects of F (flow rate) and pH of buffer on R1and R2, respectively. R1: resolution of indomethacin peak from methocarbamol peak. R2: resolution of betamethasone peak from indomethacin peak

**Fig. 4:**
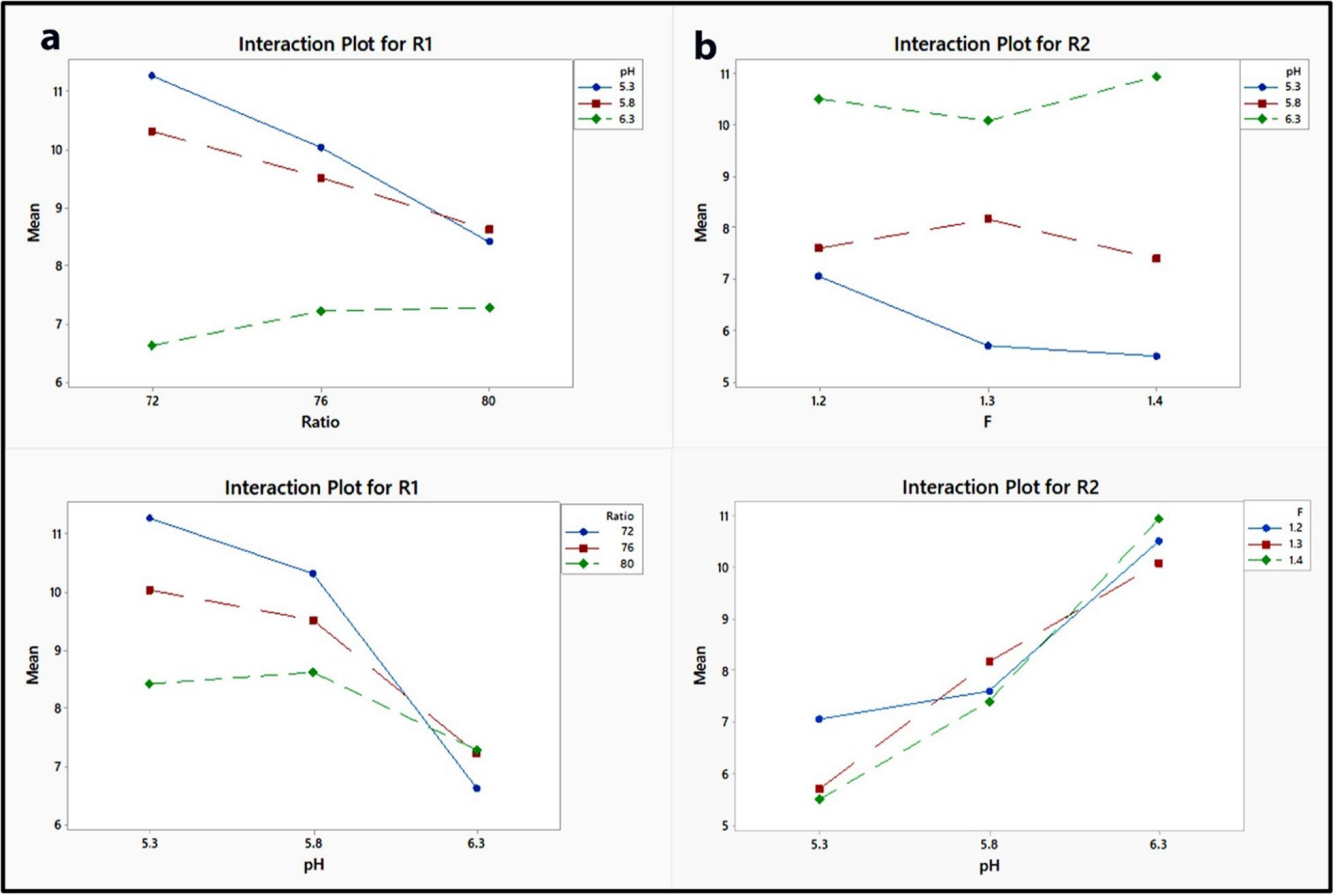
Interaction plots for the effects of pH of buffer, % of acetonitrile and flow rate on the resolution of indomethacin (IND) peak from methocarbamol (MTL) peak (R1) (**a**) and that of betamethasone (BET) peak from IND peak (R2) (**b**)

Considering the linear terms, a large coefficient of linear term for buffer pH implies that this variable dominates the other variables, whereas flow rate is more significant than the % of acetonitrile in influencing the R1 response. The positive sign of a coefficient in a regression equation indicates that the R1 response is directly related to the buffer pH and inversely related to the other two predictor variables, while the R2 response is negatively related to the % of acetonitrile and positively related to the other two predictor variables. The linear effect of pH on the R1 response is positive whereas its quadratic effect is negative, indicating that the R1 response increases as the pH increases until a critical point after which any increase in pH results in a decrease in the R1 response, and also that the linear effect of pH on the R2 response is negative whereas its quadratic effect is positive, indicating that the R2 response decreases as the pH increases from a low to a high level. Another significant effect can be deduced from the interaction between terms, where the positive sign preceding the interactive terms indicates that the two factors behave positively in the same way, i.e., to increase the response, the pH of the buffer is decreased while the percentage of acetonitrile is kept low. Furthermore, the negative value indicates that the two predictor variables behave in a negative way, i.e., in order to decrease the response, the pH of the buffer is increased while holding the flow rate at a low level. Figures 2 and 3 depict the graphical view of the two regression equations as 3D surface plots and 2D contour plots, which indicate the potential relationship between two predictor variables and the responses while maintaining the third factor constant. The non-linear impacts of the variables on responses are indicated by the curvature of the contour plots. The significant interaction between pH and % of acetonitrile was indicated by the large difference in slope between the lines, as seen in the interaction plots (Fig. 4) [53, 57]. Figure 2 shows the 3D surface plots produced by the regression models to illustrate the 3D relationship between the predictor variables and their mutual interaction with the responses. As seen in Fig. 3, the form of the contour plots reveals whether or not the interactions among the predictor variables are significant. It is clearly observed from Fig. 2a and b and Fig. 3a and b that, increasing the ratio (% of acetonitrile) from 72 to 80 at a constant F (flow rate) resulted in a decrease in R1and R2, whereas increasing the F (flow rate) from 1.2 to 1.4 at a constant ratio (% of acetonitrile) resulted in a slightly decrease in R1 and R2, but the effect of flow rate on R1 is more than R2. From Fig. 2c and d and Fig. 3c and d, an increase in ratio (% of acetonitrile) at constant pH resulted in a decrease in R1 and R2 whereas an increase in pH value from 5.3 to 6.3 at a constant ratio resulted in an increase in R2 and a slightly increased R1 to the point where further increase in pH resulted in a decrease in the R1. From Fig. 2e and f and Fig. 3e and f, it is clearly observed that the effect of pH on R1 and R2. The effects of ratio (% of acetonitrile), F (flow rate), and pH on R1 can be seen in Figs. 2a, c and e, and 3a, c and e, in which R1 decreased with increasing ratio (% of acetonitrile) and F (flow rate) from low levels to high levels, whereas R1 increased with increasing pH from a low level to a medium level of pH, at which any increase in pH leads to a decrease in R1. The effects of ratio (% of acetonitrile), F (flow rate), and pH on R2 can be seen in Figs. 2b, d and f, 3b, d and f, in which R2 decreased with increasing ratio (% of acetonitrile) and F (flow rate) from low levels to high levels, whereas R2 increased with increasing pH from a low level to a high level. Therefore, from the 3D surface and contour plots, the interaction effects between the predictor factors were significant in predicting the responses in the range of variable levels.

### Composite desirability function

**Fig. 5:**
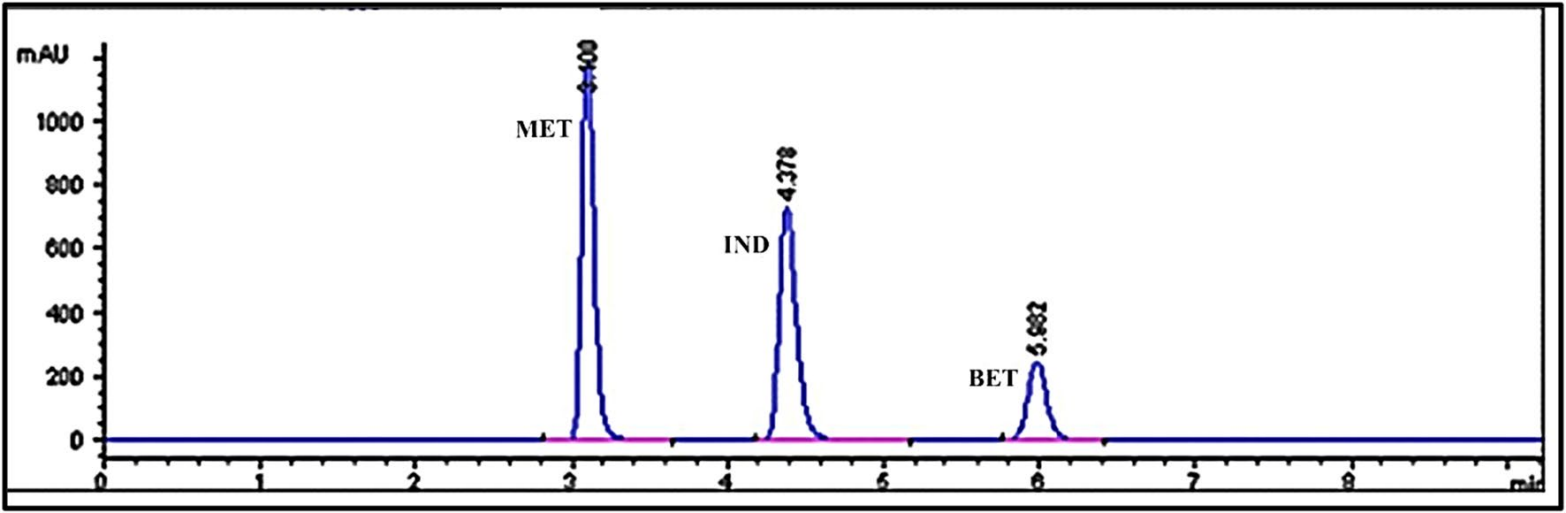
Chromatogram of MTL, IND and BET under optimum condition. (Acetonitrile = 79%, buffer pH = 5.95, Flow rate = 1.4 mL min^− 1^)

The optimization of the RP-HPLC separation was based on the following criteria: an acceptable chromatographic resolution between three peaks of the studied drugs, i.e., the goal was to get good separation between MTL and IND peaks [49] and also between IND and BET peaks (R2). Each individual response had a specific desirability (d), which was determined by defining the objective specified for each response. For each response, there were 3 objectives for selecting one of them: minimize, maximize, or target the response [58]. Each response was then given a weight that determined the shape of the desirability function. Weights must range from 0.10 to 10, with higher values indicating more significant responses and lower values indicating less significant responses. After calculating the single desirability for each response, the combined desirability (D) for all responses was obtained. A target value of 8.2 and 7.5 were selected for the R1 and R2 responses, respectively, with an importance value of 1.0 and a weight factor of 1.0. The combined desirability (D) for the optimal condition was calculated using the response optimizer tool to be 1.0 when the pH of the buffer was 5.95, the % of acetonitrile was 79.31, and the flow rate was 1.4 mL min^− 1^ [59]. Based on this statistical optimization process, the following chromatographic condition was set as the optimum: the pH of the buffer is 5.95, the % of acetonitrile is 79.31, and the flow rate is 1.4 mL min^− 1^. To make analysis of the results easier, we decided to set the % of acetonitrile at 79. The chromatogram obtained by using these conditions is shown in Fig. 5. Table 4 shows the experimental and predicted response values for the above-mentioned optimum condition. It is apparent that the response surface predictions appeared to be in good agreement with the experimental data. As a result, the BBD was effective and reliable in selecting the optimum conditions [44, 58].

**Table 4:** System suitability tests of the proposed RP-HPLC method for the simultaneous determination of MTL, IND and BET

Parameter	MTL		IND		BET	Reference value
Number of theoretical plates (N)	8821		8990		12,010	The higher the value, the more efficient the column is
Resolution factor (R)		8.54		7.52		> 2
Tailing factor (T)	0.76		0.72		0.88	≤ 2
Capacity factor (K´)	2.100		3.162		5.170	1–10
Selectivity factor (α)		1.45		1.35		≥ 1
% R.S.D. of t_R_ of 6 injections	0.77		0.50		0.79	

Where t_R_: Retention time

### Residual plots analysis

**Fig. 6:**
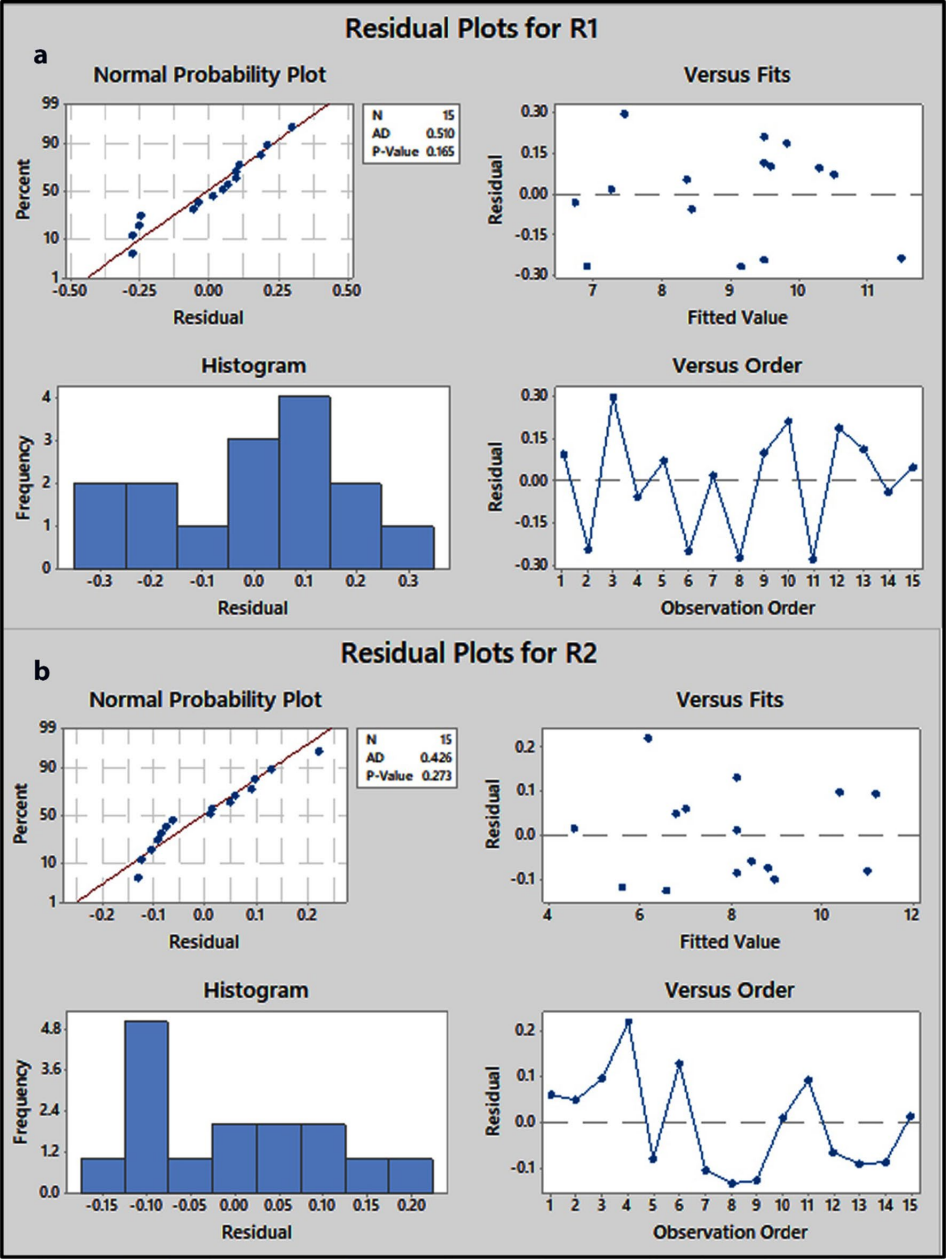
Normal probability plot, histogram, residuals versus fits and residuals versus order plots for resolution of IND peak from MTL peak (R1) (**a**) and the resolution of BET peak from IND peak (R2) (**b**)

Residual plots are used in regression and ANOVA tests to analyze the goodness of model fit and hence estimate the difficulties of a skewed distribution, outliers, and non-random error. As illustrated in Fig. 6, the residuals in the normal probability plot are normally distributed and generally produce a straight line, suggesting that outliers are absent. In plots of residuals versus fitted values and versus order of data, the residuals are randomly spread on both sides of zero, indicating that the residuals have constant variance and are uncorrelated with one another. As a result, residual analytical plots are particularly valuable in regression and ANOVA methods because they show how well a model accounts for variation in the observed data [53, 57].

### System suitability studies

After applying BBD for optimizing the chromatographic parameters and selecting the most optimal conditions, the system suitability parameters were investigated to estimate that the HPLC system was working well before the validation process. Different parameters, including R (resolution factor), T (tailing factor), N (number of theoretical plates), K′ (capacity factor), α (selectivity factor), and R.S.D. of retention times, were calculated and summarized in Table 4, and they were found acceptable.

### Method validation

By applying ICH guidelines, the proposed HPLC method was validated [47].

### Linearity

Linearity was determined in triplicates by analyzing eight different concentrations of MTL, IND, and BET. The good linearity among the peak areas and the corresponding drug concentrations was confirmed by high values of R-squared. Table 5 shows the values of slopes, intercepts, and regression coefficients for the three calibration curves and the standard deviations for each slope (Sb) and intercept (Sa).

**Table 5:** Validation parameters and results obtained by the proposed RP-HPLC method for the simultaneous determination of MTL, IND and BET

Item	MTL	IND	BET
Retention time (t_R_) (min)	3.15 ± 0.05	4.62 ± 0.31	6.17 ± 0.13
Wavelength of detection (nm)	235	235	235
Range of linearity (µg mL^− 1^)	5-600	5-300	5-300
Slope	11.549	35.756	20.242
Intercept	116.170	29.758	12.521
Regression coefficient (r^2^)	0.9994	0.9998	0.9998
LOD^**a**^ (µg mL^− 1^)	0.0966	0.750	0.672
LOQ^**a**^ (µg mL^− 1^)	0.293	2.273	2.037
Standard deviation of the slope S_b_	0.116	0.192	0.109
Standard deviation of the intercept (S_a_)	39.018	27.593	15.706
Confidence limit of the slope	11.549 ± 0.116	35.756 ± 0.192	20.242 ± 0.109
Confidence limit of the intercept	116.17 ± 39.018	29.758 ± 27.593	12.521 ± 15.706
Standard error of estimation	66.57	47.55	27.06
Intraday precision^b^	0.85-0.96-0.96	0.94-0.41-0.42	0.81-0.22-1.01
Interday precision^b^	1.54-1.67-1.17	1.21-1.20-1.86	1.77-1.53-1.83
Drug in laboratory prepared mixtures	100.41 ± 0.60	100.86 ± 0.86	100.99 ± 0.65
Drug in dosage form	99.90 ± 0.36	104.79 ± 0.29	104.89 ± 0.04
Drug added	99.70 ± 1.26	100.70 ± 1.21	100.81 ± 0.99

The interday (n = 3), average of three concentrations of (344, 430 and 516 µg mL^−1^) for MTL, (40, 50, 60 µg mL^−1^) for IND and (12, 15, 18 µg mL^−1^) for BET repeated three times in three successive days

^**a**^ Limits of detection and quantitation are determined via calculations: LOD = 3.3*SD/slope, LOQ = 10*SD/slope.

^**b**^ The intraday (n = 3), average of three concentrations of (344,430 and 516 µg mL^−1^) for MTL, (40, 50, 60 µg mL^−1^) for IND and (12, 15, 18 µg mL^−1^) for BET repeated three times within the day

### Accuracy

Six different concentrations of MTL, IND, and BET were analyzed, and each concentration was injected three times to determine the accuracy of the studied drugs in laboratory-prepared mixtures by calculating the percentage recoveries for each drug. From the accuracy results listed in Table 5, the developed method is highly accurate.

### Precision

The precision (repeatability) of intra-day was determined by using three mixtures containing 80%, 100%, and 120% of MTL-IND-BET (344-40-12 µg mL^− 1^), (430-50-15 µg mL^− 1^), and (516-60-18 µg mL^− 1^), respectively, three times. The intra-day precision, expressed in terms of % R.S.D., was found to be less than 1%. For inter-day precision, the previously mentioned concentrations in intra-day precision were analyzed three times on three successive days to estimate the day-to-day ruggedness, and the % R.S.D. of the inter-day precision was found to be less than 2%. The results of precision (intra-day and inter-day) are shown in Table 5.

### Selectivity

Selectivity ensures the developed method’s ability to distinguish and quantify the interested response of particular analyte from all other responses in the presence of interferences. Selectivity was determined by analyzing MTL, IND, and BET in laboratory-prepared mixtures containing the intact drugs at various proportions. The method’s selectivity was ensured by good separation among the peaks of the drugs, as seen in Fig. 5. In addition, the chromatograms of MTL, IND, and BET in the capsules’ samples were the same as those produced by the pure drugs, with no additional peaks observed. Furthermore, good results were achieved for the determination of MTL, IND, and BET in capsules, demonstrating the selectivity of the proposed method, as seen in Table 5.

### Limits of quantitation and detection

Limits of quantitation (LOQ) and detection (LOD) determined by the developed method were calculated using the slope of the regression line (b) and standard deviation of the intercept (Sa) according to the following relationships: LOQ = 10 Sa/b and LOD = 3.3 Sa/b, and their results are shown in Table 5. The lower the values of LOQ and LOD, the better the developed method’s sensitivity.

### Robustness

The proposed method’s robustness was tested by altering several parameters involved in chromatographic separation, as follows: pH of buffer by ± 0.2, % of acetonitrile by – 2% and flow rate by ± 0.2. The most significant response to be tested was the resolution factors (R) among MTL-IND and IND-BET peaks, which were predicted using regression model equations (Eqs. 1 and 2). Good resolution factors, as shown in Table 6, emphasize good robustness.

**Table 6:** Robustness study of the proposed RP-HPLC method using Box-Behnken experimental design

Parameter	Drugs		Normal value	pH of buffer		% of Acetonitrile	Flow rate	
				6.15	5.75	77	1.6	1.2
R1	MTL/IND	Eq-1	8.11	7.5	8.41	8.33	7.40	8.83
		Experimental value	7.98	6.66	8.13	7.72	7.61	8.25
R2	IND/BET	Eq-2	7.05	8.82	5.94	8.08	6.96	7.14
		Experimental value	7.64	9.52	6.00	8.39	6.96	7.68

R1 = resolution response between methocarbamol and indomethacin

R2 = resolution response between indomethacin and betamethasone

### Statistical analysis

The results obtained from the reversed-phase (RP)-HPLC method have been statistically compared to those obtained from the U.S.P. reference methods using the Student’s t-test and variance ratio F-test, at P = 0.05, [1]. The results showed no significant difference in accuracy and precision between the methods for each drug, as shown in Table 7.

**Table 7:** Statistical analysis of the results obtained by the proposed method and the reference methods

Statistical term	MTL		IND		BET	
	Reference method^a^	Proposed method	Reference method^a^	Proposed method	Reference method^a^	Proposed method
Mean%	99.90	100.41	100.43	100.86	100.44	100.85
S.D±	0.71	0.6	0.81	0.86	0.41	0.73
S.E±	0.29	0.25	0.33	0.35	0.17	0.30
R.S.D%	0.71	0.6	0.80	0.85	0.41	0.72
n	6	6	6	6	6	6
V^b^	0.51	0.36	0.65	0.74	0.17	0.53
t(2.57)^c^		1.33		0.70		2.29
F(5.05)^c^		0.71		1.14		3.12

No significant difference between the proposed methods and reference methods was found using the Student’s t-test and variance ratio F-test. ^a^ Reference methods for MTL, IND, and BET using HPLC methods according to USP 43-NF38 2019. ^b^ Variance, ^c^ Values in parentheses are the critical t- and F-values at P = 0.05

## Conclusion

In our study, an effective experimental design was employed for the optimization and estimation of robustness. The proposed RP-HPLC method was found to be accurate, precise, specific, rapid and simple for the separation and simultaneous determination of MTL, IND, and BET in combined dosage forms. Furthermore, the robustness of the reported method was investigated by utilizing the constructed models. Additionally, method validation was carried out in accordance with ICH requirements Q2. The proposed method can be successfully applied to quality control and routine analysis of the investigated drugs in their pharmaceutical dosage forms and laboratory-prepared mixtures with the goal of reducing laboratory waste, analysis time, and effort.

## Data Availability

All data generated or analyzed during this study are included in this published article.
